# The Combined Effects of Cr(III) Supplementation and Iron Deficiency on the Copper and Zinc Status in Wistar Rats

**DOI:** 10.1007/s12011-018-1568-7

**Published:** 2018-11-14

**Authors:** Halina Staniek

**Affiliations:** Institute of Human Nutrition and Dietetics, Poznań University of Life Sciences, ul. Wojska Polskiego 31, 60-624 Poznań, Poland

**Keywords:** Copper, Zinc, Chromium(III) propionate complex, Iron deficiency, Rats

## Abstract

The aim of the study was to assess the combined effects of chromium(III) supplementation and iron deficiency on the copper (Cu) and zinc (Zn) status in female rats. The Cr, Fe, Cu and Zn dietary and tissular levels were measured by *Atomic Absorption Spectrometry* (AAS) method. The data show that chromium(III) supplementation compensated for the negative effects of Fe deficiency on the Cu content but it deepened the effect on Zn levels in the female rats. Detailed data on the status of trace elements and their interactions in healthy subjects and patients with metabolic disorders (e.g. anaemia, diabetes mellitus) are strongly required for effective nutritional and therapeutic strategies.

## Introduction

Both iron (Fe) deficiency and its excess are manifested by health-related symptoms. Usually, deficiency or the accumulation of macro- and microelements in the organism is caused by environmental pollution, improper diet or metabolic disorders [1]. Additionally, the deficiency or accumulation of some trace elements may stimulate another pathway of their metabolism, which might induce diseases. The interaction between these elements may also stimulate many nutritional disorders [2]. Common nutritional deficiencies often include iron deficiency, which is generally related with low Fe intake, diseases, poor absorption, excessive blood loss, and increased physiological requirements as in pregnancy [3]. Iron deficiency is a common cause of anaemia. It is estimated that there are about 1 billion individuals worldwide who suffer from the effects of insufficient Fe supply [4]. Iron is a transition element that can act as an oxidant. Increased Fe accumulation influences insulin synthesis and secretion by pancreatic β cells. Fe accumulation in the liver may lead to insulin resistance by interfering with the insulin capacity to inhibit hepatic glucose production [5–9].

On the other hand, insufficient dietary Cr(III) intake was suggested as a possible risk factor of diabetes [10, 11]. Despite the fact that in 2014 the European Food Safety Authority (EFSA) published a scientific opinion in which trivalent chromium [Cr(III)] was considered a non-essential element for animals and humans [12], it is still a very popular component of supplements.

Some data indicate that Fe and Cr might compete for transferrin (Tf) binding [13–15]. However, the results of the in vivo interaction between these metals are inconsistent. Studies indicate that anaemic children had lower Cr levels than healthy subjects [16]. Also, Angelova et al. [17] showed that anaemia caused by Fe deficiency was related with reduced Cr content in the body. It suggests the possible synergic effect between Cr and Fe. Iron appears to play a particular role in the metabolism of other metals, especially copper (Cu) and zinc (Zn) [1].

Transition metals, like Fe and Cu, which have a few oxidation states, are important for the cascades of electron transfer reactions, e.g. oxygen transport, cellular respiration, free radical defence [4]. Therefore, Cu can act both as an antioxidant and pro-oxidant. Cu is an essential metal and plays an important function in metabolism [18]. Cu is involved in cellular respiration process, Fe oxidation and its cellular metabolism, peptide amidation, connective tissue and neurotransmitters biosynthesis, pigment formation (e.g. melanin) and antioxidant defence [16]. This element is a component of a significant number of redox enzymes, e.g. ceruloplasmin (Cp) and superoxide dismutase (SOD), as it monitors their proper function [19]. Moreover, multi-copper oxidase (MCO) plays a role in Fe metabolism [20]. The main Cu transporting protein is ceruloplasmin, which also acts as an enzyme that catalyses the oxidation of minerals, especially Fe [19]. Ceruloplasmin is requisite for Fe(II) oxidation into Fe(III) so that the element can be bound to its transport protein, transferrin, as well as to the main Fe storage protein, ferritin [19]. Fe deficiency anaemia may be a symptom of Cu deficiency [18]. Additionally, Cu and Fe cooperate in the reduction of oxygen in cytochrome c oxidase, which is necessary for aerobic respiration [19].

Zinc is another essential trace element engaged in numerous cellular metabolism functions. This element is involved in the activity of more than 200 enzymes. Zinc regulates the expression and activation of biological molecules such as transcription factors, channels, enzymes, adapters and growth factors along with their receptors [21]. It is involved in DNA and protein synthesis, immune function, cell division and wound healing. Zinc is responsible for proper perception of taste and smell and supports normal growth and development [19]. Zn overload in the body may cause Cu deficiency, because Zn competes with Cu in the small intestine and disturbs its absorption [15, 17]. Additionally, both Cu and Zn are components of superoxide dismutase (Cu-Zn SOD), which is a significant antioxidant in all cells. Zn oversupply promotes obesity and related diseases [22, 23]. It makes diabetic patients more susceptible to disturbances of trace elements status and glucose concentration, which is manifested by increased glycosylated haemoglobin level in the blood and severe anaemia [22, 24]. Both Zn and Cr seemed to positively affect insulin signalling, which was manifested by glucose uptake in skeletal muscles [25]. Disrupted Zn homeostasis by Zn deficiency or excessive intake affects growth, immune response, as well as neurosensory and endocrine functions [21]. For these reasons, the Zn content must be adequate to maintain essential cellular processes and biological responses [21].

Both Zn and Cu occur in large amounts in the brain hippocampus. As these elements are regarded as neurotransmitters, it is suggested that low Zn and high Cu concentrations cause attention deficit disorders, hyperactivity, behavioural disorders and depression [19]. Zn works like an antidepressant, because it induces brain-derived neurotrophic factor gene expression and increases the content Zn synaptic pool in the hippocampus [26]. It is also suggested that Alzheimer’s disease (AD) is complicated by pro-oxidant intraneuronal Fe(II) elevation as well as extracellular Zn(II) accumulation within amyloid plaque [22, 27].

Cr(III) supplementation appears to influence other elements. It causes synergistic effect on Zn levels and antagonistic one on Cu levels [28]. Therefore, it is important to maintain appropriate dietary balance of trace elements, such as Fe, Zn, Cu and Cr. It seems that the metabolism of Fe, Cr(III), Cu and Zn is interrelated and these elements may directly or indirectly cause the development of various disorders, e.g. diabetes, anaemia, depression. Some researchers recommend measuring the content of other trace elements along with the indices of Fe homeostasis in case of Fe deficiency anaemia. They suggest that the correction of the trace element status in Fe deficiency may help to increase possible synergistic interactions or reduce antagonistic ones [3]. We hypothesised that Cr(III) supplementation alleviated the influence of Fe deficiency in the diet on the Cu and Zn status. Therefore, we investigated the combined effects of dietary Cr(III) propionate complex supplementation and Fe deficiency on the Cu and Zn status in animal model.

## Material and Methods

### Test Chemicals

Iron(III) citrate (contained 16.6% Fe) was purchased from Sigma-Aldrich, USA. The chromium(III) complex with propionic acid (Cr3), in the form of nitrate salt [Cr_3_O(O_2_CCH_2_CH_3_)_6_(H_2_O)_3_]NO_3_, was synthesised in the laboratory at the Department of Technology and Instrumental Analysis (Poznań University of Economics) by the procedure described in detail by Earnshaw et al. (1966) [29]. The Cr3 contained 21% of Cr determined by the AAS method (spectrometer AAS-3 with background correction, Zeiss, Germany).

### Animal and Diets

The 36 6-week-old ♀ Wistar rats were purchased from the Department of Toxicology, Poznań University of Medical Sciences, Poland. All applicable international, national and/or institutional guidelines for the care and use of animals were followed. All procedures performed in studies involving animals were in accordance with the ethical standards of the institution or practice at which the studies were conducted. The study was approved by the Local Animals Bioethics Committee No. 60/2013. The rats had been acclimated to the laboratory conditions for 5 days before the study began. Then, the rats were divided into six groups of roughly equal initial mean body weight 130.5 g. The animals were housed in single cages, at controlled temperature, air humidity (19–22 °C, 55–60% of ambient air humidity). The light and the dark cycles in the experimental room were 12 h each. All groups were fed diets semi-purified AIN-93M [30] (Table 1), modified according to the two-factorial experimental model for 6 weeks. The rats were allowed free access to food and distilled water throughout the whole experiment.

**Table 1:** The chemical composition of experimental AIN-93 diets modified with Fe(III) and Cr(III) contents (mean ± SD)

Component	Unit	Content of compound in experimental diets					
		C1	C50	C500	H1	H50	H500
Energy	MJ 100 g^−1^	1.82 ± 0.00	1.83 ± 0.03	1.89 ± 0.06	1.88 ± 0.04	1.82 ± 0.03	1.81 ± 0.02
Fat	%	7.46 ± 0.05	7.22 ± 0.08	7.26 ± 0.31	7.31 ± 0.10	7.11 ± 0.06	6.83 ± 0.05
Protein	%	17.12 ± 0.10	17.17 ± 0.14	17.27 ± 0.24	17.42 ± 0.30	17.01 ± 0.12	17.72 ± 0.39
Carbohydrates	%	63.46	63.67	63.45	64.05	63.11	64.77
Dry mass	%	90.47 ± 0.05	90.54 ± 0.22	90.26 ± 0.08	89.99 ± 0.36	89.67 ± 0.54	89.90 ± 0.23
Ash	%	2.47 ± 0.04	2.48 ± 0.11	2.32 ± 0.50	2.36 ± 0.21	2.67 ± 0.08	2.58 ± 0.08
Fe	mg kg^−1^	58.05 ± 0.70	57.09 ± 2.83	59.13 ± 1.98	218.12 ± 7.53	207.55 ± 21.49	229.78 ± 13.04
Cr	mg kg^−1^	1.24 ± 0.23	50.04 ± 6.48	425.14 ± 10.28	1.95 ± 0.61	49.42 ± 5.40	431.59 ± 14.82
Ca	g kg^−1^	4.96 ± 0.13	5.16 ± 0.13	5.02 ± 0.11	5.17 ± 0.02	5.09 ± 0.11	4.94 ± 0.17
Mg	mg kg^−1^	441.42 ± 8.99	473.12 ± 1.44	511.73 ± 17.55	503.86 ± 23.80	501.62 ± 19.90	529.98 ± 15.50
Zn	mg kg^−1^	52.51 ± 1.60	50.71 ± 1.90	52.41 ± 2.02	44.80 ± 4.21	40.86 ± 0.49	43.13 ± 3.00
Cu	mg kg^−1^	5.45 ± 0.94	4.43 ± 0.15	5.93 ± 0.79	5.82 ± 0.71	6.28 ± 0.20	6.62 ± 1.02

The study was carried out on 36 healthy female Wistar rats, which were divided into 6 investigational groups (6 animals in each) with different Fe contents in the diets [deficient (D) 5 mg kg^−1^, 10% RDA, adequate (C) 45 mg kg^−1^, 100% RDA). At the same time, they were supplemented with Cr(III), given as Cr(III) propionate complex, at doses of 1, 50 and 500 mg kg^−1^: Control C1 Fe 45 mg kg^−1^, Cr 1 mg kg^−1^; Group C50 Fe 45 mg kg^−1^, Cr 50 mg kg^−1^; Group C500 Fe 45 mg kg^−1^, Cr 500 mg kg^−1^; Group D1 Fe 5 mg kg^−1^, Cr 1 mg kg^−1^; Group D50 Fe 5 mg kg^−1^, Cr 50 mg kg^−1^; Group D500 Fe 5 mg kg^−1^, Cr 500 mg kg^−1^.

The food intake was registered daily at the same time, while body weight gains were monitored weekly.

### Data Collection

At the end of the experimental trial, after 12-h starvation, the animals were euthanised by asphyxiation with CO_2_. Blood was collected into tubes; tissue samples (heart, kidneys, liver, spleen, pancreas, ovaries) were harvested, weighed and frozen at − 20 °C.

### Laboratory Analyses

Chemical composition of diets was determined based on the following assays: the protein content was determined by the Kjeldahl method and the fat content was measured by the Soxhlet method. The ash content was determined by burning diet samples at 550 °C. The content of carbohydrates was determined from the difference according to the equation, carbohydrates = 100 − (water + protein + fat + ash).

The diet and tissue samples for mineral analyses were digested with concentrated 65% spectra pure nitric acid (Merck) in a Microwave Digestion System (MARS-5, CEM, USA).

The tissue samples (kidney, liver, spleen, heart and femur) at mass 0.5–1.5 g were digested with 5 ml concentrated (65%) spectra pure HNO_3_ (Merck) in Teflon pressure vessels. Thereafter, having diluted the samples to the measuring range for given element in deionised water, the analysed elements concentrations in the analytes were measured. The concentration of copper (Cu), zinc (Zn) and iron (Fe) in mineralised samples was determined with the flame atomic absorption spectrometry method (F-AAS) (Zeiss AAS-3, with background correction, Germany).

The accuracy of Cu and Zn measurements was assured by simultaneous analysis of certified reference material (Pig Kidney BCR No. 186, Brussels), The mean recoveries of certified levels (expressed as percentage of mean certified values) were as follows: Cu 101%, Zn 98%.

### Statistical Analysis

The data are expressed as mean ± SD. The results were analysed with two-way analysis of variance (two-way ANOVA/MANOVA, factors of Fe and Cr dietary levels, test F). If the two-way ANOVA indicated a significant Fe(III) level effect, Cr supplementation effect, or Fe × Cr interaction, a subsequent one-way ANOVA and post hoc comparison were performed using Tukey’s test. Statistical significance was considered at *p* < 0.05. Statistical tests were performed using Statistica version 12.0 for Windows (StatSoft, Poland).

The data were expressed as mean ± SD. The results were analysed with two-way analysis of variance (two-way ANOVA/MANOVA, factors of Fe and Cr dietary levels, test F). If the two-way ANOVA indicated a significant Fe(III) level effect, Cr supplementation effect, or Fe × Cr interaction, a subsequent one-way ANOVA and post hoc comparison were made using Tukey’s test. The statistical significance limit was *p* < 0.05. The Statistica 12.0 software for Windows (StatSoft, Poland) was used for statistical tests.

## Results

Tables 2 and 3 show the main effects of different Fe(III) levels in the diet (factor A) and Cr3 supplementation (factor B) (for each factor: A and B, independently of the other factor). Table 4 presents the interactive effects of both factors (the combination of A and B factors) on the tissular Cu and Zn contents in rats.

**Table 2:** The main effects of different Fe(III) levels in the diet and Cr(III) supplementation on the tissular Cu contents in female rats

Parameters	Main effect					
	Factor A Fe(III) level in diet			Factor B Cr(III) level in diet		
	Fe(III) level in diet deficient vs. control (mg kg^−1^ diet)		*p*-value	Cr(III) level in diet 1 vs. 50 vs. 500 (mg kg^−1^ diet)		*p*-value
Liver Cu content (μg g^−1^ d.m.)	5 (deficient) 45 (control)	27.505 ± 2.410 29.234 ± 3.491	NS	1 (control) 50 (I level) 500 (II level)	29.799 ± 1.676 27.677 ± 3.971 27.460 ± 2.202	NS
Kidney Cu content (μg g^−1^ d.m.)	5 45	46.547 ± 12.505 48.380 ± 11.100	NS	1 50 500	43.791 ± 3.504 53.885 ± 14.594 39.838 ± 13.015	NS
Spleen Cu content (μg g^−1^ d.m.)	5 45	8.333 ± 1.502^b^ 6.611 ± 1.057^a^	*p* < 0.01	1 50 500	7.251 ± 1.004 8.145 ± 1.873 7.197 ± 1.587	NS
Heart Cu content (μg g^−1^ d.m.)	5 45	22.561 ± 2.364 20.410 ± 2.800	NS	1 50 500	21.512 ± 2.582 20.953 ± 3.208 22.604 ± 2.259	NS
Femur Cu content (μg g^−1^ d.m.)	5 45	4.442 ± 0.562 4.021 ± 0.396	NS	1 50 500	4.324 ± 0.424 4.253 ± 0.563 4.182 ± 0.646	NS

*The values which do not have the same superscript letter are significant differently (two-way analysis of variance, *p* < 0.05); *NS* no significant effect, *d.m.* dry mass

**Table 3:** The main effects of different Fe(III) levels in the diet and Cr(III) supplementation on the tissular Zn contents in female rats.

Parameters	Main effect					
	Factor A Fe(III) level in diet			Factor B Cr(III) level in diet		
	Fe(III) level in diet deficient vs. control (mg kg^−1^ diet)		*p*-value	Cr(III) level in diet 1 vs. 50 vs. 500 (mg kg^−1^ diet)		*p*-value
Liver Zn content (μg g^−1^ d.m.)	5 (deficient) 45 (control)	107.505 ± 7.611 114.201 ± 10.759	NS	1 (control) 50 (I level) 500 (II level)	108.249 ± 10.587 111.736 ± 11.129 111.384 ± 7.817	NS
Kidney Zn content (μg g^−1^ d.m.)	5 45	105.045 ± 19.432 107.349 ± 13.214	NS	1 50 500	113.164 ± 16.849 109.390 ± 12.526 97.199 ± 17.368	NS
Spleen Zn content (μg g^−1^ d.m.)	5 45	79.337 ± 11.631^a^ 87.618 ± 9.133^b^	*p* < 0.01	1 50 500	92.900 ± 10.105^b^ 82.242 ± 9.519^a^ 76.315 ± 8.150^a^	*p* < 0.001
Heart Zn content (μg g^−1^ d.m.)	5 45	73.002 ± 10.190^b^ 63.285 ± 7.671^a^	*p* < 0.01	1 50 500	67.727 ± 9.738 64.030 ± 9.273 73.562 ± 10.103	NS
Femur Zn content (μg g^−1^ d.m.)	5 45	326.884 ± 23.148 322.175 ± 24.714	NS	1 50 500	329.018 ± 20.021 330.378 ± 17.442 315.563 ± 30.064	NS

*The values which do not have the same superscript letter are significant differently (two-way analysis of variance, *p* < 0.05); *NS* no significant effect, *d.m.* dry mass

**Table 4:** The combined/interactive effects of different Fe(III) levels in the diet and Cr(III) supplementation on the Cu and Zn content in the tissues of female rats (mean ± SD)

Parameters	Experimental groups					
	D1	D50	D500	C1 (control)	C50	C500
Liver Cu content (μg g^−1^ d.m.)	30.082 ± 1.716	25.426 ± 1.663	27.526 ± 0.735	29.421 ± 1.907	30.490 ± 4.402	27.372 ± 3.704
Kidney Cu content (μg g^−1^ d.m.)	43.791 ± 3.504	53.885 ± 14.594	39.838 ± 13.015	53.287 ± 11.761	46.815 ± 3.590	45.561 ± 18.544
Spleen Cu content (μg g^−1^ d.m.)	7.750 ± 0.640	9.282 ± 1.432	7.731 ± 1.854	6.586 ± 1.117	6.724 ± 1.333	6.485 ± 1.033
Heart Cu content (μg g^−1^ d.m.)	21.732 ± 3.625	22.446 ± 2.164	23.534 ± 0.873	21.218 ± 0.240	19.088 ± 3.583	21.364 ± 3.184
Femur Cu content (μg g^−1^ d.m.)	4.408 ± 0.551	4.534 ± 0.577	4.360 ± 0.701	4.255 ± 0.255	3.902 ± 0.321	3.944 ± 0.607
Liver Zn content (μg g^−1^ d.m.)	103.954 ± 9.807	105.942 ± 4.551	112.619 ± 5.862	115.407 ± 8.772	117.530 ± 13.192	110.148 ± 9.965
Kidney Zn content (μg g^−1^ d.m.)	109.089 ± 20.089	112.861 ± 16.556	93.185 ± 18.438	119.956 ± 3.943	105.919 ± 6.988	101.214 ± 17.276
Spleen Zn content (μg g^−1^ d.m.)	91.240 ± 8.953	76.040 ± 6.236	71.031 ± 8.803	95.677 ± 13.369	88.808 ± 7.943	81.600 ± 1.460
Heart Zn content (μg g^−1^ d.m.)	69.768 ± 11.816	69.032 ± 8.653	80.207 ± 7.158	64.325 ± 4.990	59.027 ± 7.487	66.918 ± 8.250
Femur Zn content (μg g^−1^ d.m.)	333.524 ± 22.098	329.860 ± 21.273	317.269 ± 27.436	321.507 ± 17.056	330.895 ± 15.208	313.856 ± 35.689

* D, Fe-deficient groups (5 mg kg^−1^ diet); C, Fe control groups (45 mg kg^−1^ diet), supplemented with Cr(III) at doses of 1, 50 and 500 mg kg^−1^, respectively

The Fe-deficient diet significantly increased the spleen Cu content (*p* < 0.01) by 26% in comparison with the control Fe level in the diet (Table 2). The different Fe(III) level in diet did not affect the Cu content in the rats’ livers, kidneys, hearts or femurs. However, it was observed some trends namely the Fe-deficient diet increased the Cu levels in the heart and femur. However, the opposite trend was observed in the hepatic and renal Cu content. The tissular Cu contents were not affected by the Cr(III) level in the diet (Table 2). The dietary Cr3 supplementation tended to decrease the hepatic and femur Cu levels as the Cr(III) dose increased. Cr(III) supplementation at a dose of 50 mg kg^−1^ diet increased the Cu content in the kidneys and spleen, but a dose of 500 mg kg^−1^ diet reduced these indices to the same or lower level than in the control group. The two factors had no interactive effect on the Cu status (Table 4).

As far as the tissular Zn levels in rats are concerned, the research showed that the Fe-deficient diet decreased the spleen Zn content (*p* < 0.01) by 9.4%, but increased the heart Zn level (*p* < 0.01) by 15.4%, as compared with the control group (Table 3). The Fe-deficient diet tended to decrease the hepatic and renal Zn contents, as compared with the recommended Fe(III) level, but the differences were not statistically significant. The Cr(III) level in the diet had no effect on the Zn content in the liver, kidney, heart and femur, except the spleen Zn level (*p* < 0.001). Cr(III) supplementation at doses of 50 and 500 mg kg^−1^ diet reduced the spleen Zn content by 11.5% and 17.8%, respectively, as compared with the control group. There were no significant interactive effects of the experimental factors on the tissular Zn levels (Table 4).

## Discussion

In developed countries, dietary supplementation is a widespread strategy preventing mineral deficiencies [31]. However, food ingredients and supplements may interact with each other. An increasing number of investigations showed a possible relation between Fe(III) and Cr(III) [10, 16, 32, 33]. In this study dietary supplementation with the Cr3 complex did not affect the Fe status, but increased the Cr content in tissues. However, this influence was weaker in the Fe-deficient groups than in those with the control Fe level. Like other authors [16, 17, 34, 35], we also noticed that the Fe deficiency lowered the Fe tissular content, but simultaneously the Cr3 supplementation mitigated these symptoms of Fe deficiency [36]. In turn, in another study the Fe(III) oversupply given separately or in combination with Cr(III) decreased the Cr content in female rats’ serum, liver and kidneys. Moreover, it caused interaction because the supplementary Cr3 complex increased the kidney Fe level in groups with Fe excess, as compared with those with an adequate Fe level in the diet. Although, the doses of supplementary Cr(III) did not change most of the biochemical, haematological and tissular parameters of Fe metabolism [37].

Reports show that transferrin, which is the main Fe transport protein in the bloodstream, transfers Cr(III) in vivo as well. This may indicate interaction between these elements. According to Deng et al. [33], Cr(III) ions bind to the two Fe(III)-binding sites of apotransferrin. As the metal binding sites are divided between Fe(III) and Cr(III) ions, excess Fe(III) reduces the ability of Cr(III) to bind to transferrin (Tf) [33]. It was shown that the physiologically appropriate concentration of Cr(III) did not affect the the binding of Fe(III) at physiological concentrations to Tf [14, 33]. However, in the presence of bicarbonate the conformation changed and Cr(III) ions bound to transferrin rapidly and tightly [32]. Deng et al. [33] also noticed that keeping transferrin in the presence or absence of glucose caused conformation changes, which influenced the binding of Cr(III) ions to the protein in vitro. The authors explained that after binding Cr(III) ions tightly, glycated transferrin adopted a different conformation than freshly dissolved, non-glycated transferrin. Moreover, the researchers observed that heat storage alone caused Cr(III) ions to bind tightly in only one of the two metal binding sites. These modifications decrease the transferrin capacity to transport Cr(III) ions in vivo in rodents [33]. This might partly explain the lower Cr levels in diabetic subjects and indicate an antagonistic effect between Cr(III) and Fe(III). In our previous studies, there was a higher transferrin level in Fe-deficient rats but the rats fed with an excessive Fe content in the diet did not differ from the healthy ones [36, 37].

It seems that the metabolism of Fe, Cr(III), Cu and Zn are interrelated and these elements may play an important role in particular, in the development of diabetes or anaemia. Insufficient dietary Cr(III) intake might be a possible risk factor in the development of diabetes [10, 11]. It is difficult to document chromium deficiency because of analytical problems caused by very low levels of this element in the blood and tissues [2]. For this reason, there are more and more doubts about the essentiality of Cr(III). Since 2014, the European Food Safety Authority (EFSA) has not recognised Cr as an essential element for animals and humans [38].

Several studies showed that Cr(III) supplementation influenced the Cu and Zn status [10, 39–42]. Earlier studies indicated that the Cr(III) propionate complex (Cr3) increased the liver and spleen Cu content as well as the kidney Zn content at doses of 20 and 100 mg Cr kg^−1^ b.m. But during a 4-week experiment, the healthy female rats’ liver and spleen Zn contents as well as the renal Cu content were not affected [39]. This is partially similar to the results of this study. In another study, we noted that the dietary exposure of pregnant rats to the Cr3 complex at repeated doses of 7.2 mg Cr kg^−1^ b.w day^−1^ increased the maternal liver and kidney Cr levels and decreased the hepatic Cu and Zn contents by 9% and 12%, respectively. However, it did not affect the maternal Fe status or foetal Cr and Fe levels, while it increased the foetal liver Zn level and decreased the kidney Cu content [40].

On the other hand, there are a lot of reports on the beneficial effects of different Cr(III) compounds in diabetes [43–46]. Hyperglycemia may be related with lower plasma Cr concentration and increased urinary Cr excretion. Cr treatment applied to diabetic patients had beneficial effects on glycemic control [11]. Basaki et al. [11] demonstrated that there were no significant changes in the Fe concentration between diabetic and control subjects, but the serum Cr, Zn and Cu concentrations were lower in the diabetic patients than in the control. Therefore, diabetes mellitus can interfere with the homeostasis of trace elements. According to some reports, the Cr contents in the urine, hair and other tissues or body fluids does not always reflect the Cr status [2]. Doukan et al. [10] reported that dietary chromium histidinate (CrHis) supplementation affected the serum and tissular Cr, Zn, Se, Mn and Cu contents in diabetic rats. The authors noted that after 10 weeks CrHis supplementation increased the Cr and Zn contents in the liver, kidney, and serum, but decreased the Cu levels both in diabetic and non-diabetic rats. However, it did not change Fe levels in either group [10]. In another study, we also observed that the liver Fe, Zn, Cu and Mg contents in obese Zucker rats (an early stage diabetes model) were lower than in ZDF (a type 2 diabetes model) and lean Zucker rats (an insulin resistance model) [41].

Vlizlo et al. [28] observed that Cr(III) supplementation had synergistic effect on Zn levels and antagonistic effect on Cu levels. Also, the results obtained by Sahin et al. [42] suggested that Cr (Pic)_3_ at doses of 200–1200 μg Cr kg^−1^ increased the Cr and Zn content, but decreased the Cu level in Japanese quails’ serum, liver, kidneys and muscles. However, during 93 days of the experiment the serum and tissular Fe contents did not change as the Cr(III) content in the animals’ diet increased [42].

Another study [47] showed that a diet enriched with chromium yeast at a dose of 0.5 mg kg^−1^ given to hens for 2 months increased their Cr and Cu levels, but did not affect the Zn content in their liver. Similarly, Amatya et al. [48] observed that broilers supplemented with chromium yeast and CrCl_3_ at a dose of 200 μg Cr kg^−1^ for 21 and 35 days had higher Cu content in the blood serum and liver. Dallago et al. [49] experimented on lambs and noted a positive linear correlation between the dose administered and Cr accumulation in the heart, lungs and testis after an 84-day treatment with CrPic at the following doses: placebo, 0.250, 0.375 and 0.500 mg day^−1^. Additionally, urinary excretion of Cr took place in a time- and dose-dependent fashion. However, CrPic given orally did not significantly affect the Cr content in other tissues, i.e. liver, kidney, spleen, lymph node, skeletal muscle and bone.

Liu et al. [50] conducted a 42-day experiment on chickens and proved that the exposure to different doses of chromium(III) chloride (CrCl_3_) in drinking water 0, 1/8, 1/4 and 1/2 LD_50_ (i.e. 0, 625, 1200 and 2500 mg kg^−1^, respectively) could disrupt the absorption and deposition of other trace elements in the brain and serum. The long-term exposure of the chickens to high Cr(III) doses in drinking water increased the Cr content in their serum and brains. The authors observed that CrCl_3_ exposure decreased the Cu content in the serum and brain, increased the Fe and Zn contents in the serum and decreased the Fe and Zn contents in the brain. They concluded that the blood-brain barrier may prevent the accumulation of these elements in the brain exposed to CrCl_3_. There were opposite effects of CrCl_3_ supplementation on the Fe content in the serum and brain. This, in turn, may affect the brain function, e.g. cognitive capacity. Impaired glucose metabolism due to insulin resistance may cause memory impairment [51]. The supplementation of rats with chromium(III) glycinate (CrGly) and chromium(III) acetate (CrAct) improved insulin action and alleviating memory acquisition in a dose-dependent manner [51]. The researchers observed that the average memory time spent in the target quadrant (MPTISITQ) was shortened and the spiral memory acquisition phase (SMAP) was extended by restoring the tissue Cr reserve and improving GLUTs expressions in these rodents’ brain. Dubey et al. [52] demonstrated that treatment with chromium picolinate (CrPic) produced significant antidepressive effect. It was evidenced by decreased immobility time in a modified forced swimming test (FST) during depression induced in rats by chronic unpredictable mild stress (CUMS). There have been suggestions that Zn deficiency is the cause of mood disorders and depression [53]. Recent research showed the important role of Zn in the pathophysiology and treatment of affective disorders and it emphasised the potential value of Zn as a marker of these diseases. The reduced Zn status in the body induces somatic and neurological symptoms such as rough skin, growth retardation, neurosensory disorders as well as psychopathological symptoms, which resemble depression symptoms, e.g. reduced sense of taste, poor appetite, reduced immune function, cognitive impairment, irritability, mood lability [26]. Supplementation with zinc compounds was recommended to female students to combat depression [53]. Some specialists consider that high Cu levels, especially in the case of zinc deficiency, may cause various medical conditions such as fatigue, muscle and joint pain, headaches, premenstrual syndrome, hypertension, hyperactivity, depression, insomnia, autism and schizophrenia [19]. Fe deficiency is also considered a cause of depression, especially in pregnancy [54], which may be the result of interactions between Zn and Fe. Bjørklund et al. [3] indicated both synergistic and antagonistic interactions between Fe and Zn, especially in terms of Fe deficiency and supplementation, which affect the zinc status in the human body. The antagonistic effect of Zn on the Fe status may result from competitive binding to metal transporters. Also, Zn may be engaged in hepcidin regulation. Zn is bound to and transported by albumin in the plasma at about 60% and by transferrin at about 10%. As transferrin also transports Fe, excessive amounts of this element may inhibit Zn absorption, and vice versa.

It was observed that food fortification with Fe in Fe-deficiency anaemia did not influence Zn absorption significantly. It can be explained by the fact that Zn can be held in metallothionein stores and it can also be transferred by metal transporters such as ZnT and ZIP [19]. On the other hand, Zn overload considerably reduces Cu absorption because metallothioneins absorb both of these elements [19]. Zn excess in the organism may cause Cu deficiency because Zn may competitively inhibit the gastrointestinal absorption of Cu [22]. High doses of supplemental Zn taken over long periods of time may reduce Cu binding in the intestine and cause Cu deficiency with related anaemia [19, 22]. Excessive Zn absorption was observed to decrease Cu and Fe absorption [19]. However, Herrera et al. [55] suggest that transferrin does not play a primary role in the distribution of Mn, Cu and Zn to tissues.

Research on animals showed that Fe deficiency affected the status of Fe, Zn, Cu, Mn, Mg and Ca. Yokoi et al. [35] found that the Fe content decreased in most of the tissues tested in male Wistar rats. However, the Cu concentrations in the liver, spleen, tibia and blood increased, whereas the Cu level in the femoral muscle decreased. Moreover, the Zn concentration in the blood was reduced, but had no effect on the tissular Zn content in the Fe-deficient rats, as compared with the control rats [35].

On the other hand, Vaz et al. [56] studied male Wistar rats for 33 days and found lower Cu absorption both in Fe-deficient and control animals after supplementation (addition) with inulin-type fructans (ITF). Furthermore, Fe deficiency also impaired Zn absorption, irrespective of the presence of ITF in the diet [57].

In turn, Gutowska et al. [1] found that the rising levels of Zn and Cu resulted in a loss of Fe in roe deer’s bone. The highest content of Fe was noted at low levels of Ca and Cu or Zn and Cu [1]. The Fe and Cu contents in roe deer’s bones were positively correlated, but Fe was negatively correlated with Zn irrespective of the Cu content. While, the Cu content had no effect on the positive correlation between Fe and Cr [1]. The authors observed that the accumulation of Cr concurrently with high levels of Ca was accompanied by the accumulation of Fe. This fact revealed relationships between Cr, Cu and Fe. The accumulation of Cu at low levels of Fe had no effect on the Cr content. Increased Fe content was accompanied by a loss of Cr. They noted that the Cr content was the highest at low Cu concentrations and high Fe levels in roe deer’s mandibles [1].

The Fe and Cu intake with drinking water raised the contents of these metals in rats’ hair and led to Fe accumulation in the adipose tissue for 3 months, whereas the Cu content in the adipose tissue did not change [58]. Moreover, these elements lowered the Cr adipose content [59]. In another study, simultaneous administration of Cr and V respectively increased the hepatic Fe content and the renal Zn level 1.7 and 1.45 times, as compared with the control group [60].

Shah et al. [16] observed that the Fe and Cr contents in the hair, blood and urine of anaemic boys and girls aged 1–5 and 6–10 years were lower than in the control subjects. The patients’ Zn and Cu concentrations in the tested biological samples were also reduced, but the anaemic children had higher Cu content in urine than the healthy children. Fe and Cu deficiency contributes to the development of anaemia. Copper is vital for the function of numerous enzymes, including ceruloplasmin, which is responsible for Cu transport, antioxidative protection and Fe metabolism [16]. It is suggested that anaemia is related to defects in Fe mobilisation, which is caused by the combined defect of ceruloplasmin ferroxidase activity and intracellular utilisation [16]. Some studies showed that the Cu level in Fe deficiency anaemia was higher than in non-iron deficiency anaemia and that it was also higher in non-anaemic pregnant women than in healthy non-pregnant women. But, the Zn concentration in Fe-deficient anaemic pregnant women was lower than in the other groups [61, 62]. According to Osredkar reports, this fact can be explained by competitive inhibition of the absorption of some metals in the gastrointestinal tract, or it might be caused by changes in hormone levels, e.g. oestrogen and insulin during pregnancy [19]. It was noticed that pregnant women with Fe deficiency anaemia had reduced serum Fe, serum ferritin and Fe saturation, but higher serum TIBC and Cu concentrations than non-anaemic pregnant women [61]. Fe deficiency caused an increase in the hepatic Cu content. Pregnant women’s intestinal Fe absorption may be reduced due to a high serum Cu level [61]. McArdle et al. [63] suggested that it was related with increased serum Cu concentrations and ceruloplasmin activity in the maternal serum. It indicated the antagonistic Cu-Fe interaction in pregnant women with Iron-Deficiency Anaemia (IDA) [61].

The results of comparisons of the serum Zn and Cu levels between patients with iron-deficiency anaemia (IDA) and non-anaemic subjects are ambiguous. These observations confirm the NHANES II data, which indicated that both low and high serum Cu concentrations were positively related with anaemia [64]. Most data showed that IDA patients had reduced serum Zn concentration than iron-adequate subjects [16, 17, 65]. However, some researchers did not observe significant changes in Zn and Cu concentrations between IDA patients and healthy subjects [66]. There were suggestions that Fe deficiency may be associated with the low Zn level and high Cu level in anaemic subjects. Zn and Fe supplementation was proposed to correct iron deficiency anaemia [65].

Some studies demonstrated that anaemic patients had lower Cr concentrations in blood than healthy subjects [16], which indicated antagonism between Cr(III) and Fe(III) in binding to apotransferrin [16, 67]. This is also confirmed by the results of this current study. Lukaski et al. [68, 69] suggested that high-dose and long-term Cr(III) supplementation may adversely affect Fe metabolism in adults. Furthermore, several studies also showed an association between between iron metabolism and diabetes [5, 8, 9, 70]. One of the proposed mechanisms is oxidative stress induced by increased iron deposition in the liver and β cells. It causes cell damage and insulin resistance, it disorders insulin secretion and dysregulates glucose [71].

Other authors found that menopause significantly affected insulin metabolism and the fasting plasma glucose concentration. It suggests that menopause increases the risk of diabetes. Moreover, like diabetes, menopause also causes abnormal metabolism of trace elements [72].

According to some studies comparing premenopausal and postmenopausal women, the latter had higher serum Fe level, lower serum Cu level and unchanged Zn level [73, 74]. Skalnaya et al. [72] observed changes in the status of trace elements in postmenopausal women with prediabetes and type 2 diabetes. The most prominent changes were detected in Fe and Cu metabolism, while the Zn status was not affected [72]. It was noted that the postmenopausal women with diabetes had significantly higher serum Fe, Cu and Zn concentrations than the healthy subjects [72]. The serum ceruloplasmin concentrations were significantly increased in prediabetes, but such changes were not observed in the diabetic postmenopausal women [72]. The serum Fe and Cu levels tended to increase along with glycated haemoglobin Hb (HbA_1C_) concentration. The serum ferritin levels in the diabetic women were higher than in the control subjects, but Tf saturation was decreased. Moreover, dysregulated Cu homeostasis in diabetes may also exacerbate altered Fe balance due to the influence of Cp on Fe transport [72]. For this reason, the pro-oxidative effect of Fe and Cu is suggested as a mechanism contributing to diabetes [72]. In this experiment, we observed that the Fe deficiency increased the rats’ TIBC and transferrin levels but reduced their ferritin level and most haematological parameters. However, the simultaneous addition of high doses of Cr3 did not deepen these adverse changes [36]. Moreover, Chen et al. [20] showed that 4-week CrPic supplementation improved some element metabolism disorders in BALB/c mice with PolyCystic Ovary Syndrome (PCOS). The PCOS control group had lower serum, muscle, and bone Cr levels than the PCOS group supplemented with Cr(III) and healthy mice. The serum, liver and bone Fe contents in the PCOS group were higher than in the control group. These data suggested that Cr supplementation affected the Fe level in the PCOS mice but it did not cause Fe deficiency when compared with the control group. There were positive correlations (*r* = 0.61) between the Cu/Zn ratio and fasting insulin in all the mice [20].

Vieyra-Reyes et al. [75] proved that both Wistar rats’ age and sex significantly affected the intensity and course of oxidative stress induced by Fe deficiency. The results received by Bay et al. [34] demonstrated significantly lower serum Se and Zn levels. Also, the antioxidative capability and oxidative status were changed in pica and Fe-deficiency anaemia groups, as compared with the control group. Vlizlo et al. [28] showed that a diet with Cr(III) supplementation could strengthen the main mechanisms of lipid peroxidation defence by stimulating the enzymatic activity of the antioxidative system and natural resistance factors. These authors reported that Cr(III) at doses of 70 or 140 μg dm^−3^ decreased the activity of SOD, but increased the activity of catalase (CAT), glutathione peroxidase (GPx) and glutathione reductase (GR) in rats [28]. Whereas, the concentration of thiobarbituric acid reactive substances (TBARS) was 20.3% lower than in the control group. In turn, Surdaram et al. [76] observed lower SOD, GR and CAT activities in the liver of diabetic rats. Moreover, chromium picolinate (CrPic) administered orally at a dose of 1 mg kg^−1^ b.w. for 4 weeks had advantageous effect as it normalised the rats’ glucose levels, lipid peroxidation and antioxidative status.

The individual and combined Cr and Zn supplementation improved the antioxidative status in patients with type 2 diabetes mellitus [77]. Both Zn and Cr seemed to have beneficial influence on insulin signalling in skeletal muscles as they improved glucose uptake [22, 25]. Sadri et al. [44] showed that the combination of leucine, Zn and Cr had beneficial effect on diabetic Wistar rats’ blood glucose level, while supplementation with Zn-Cr, Leu-Zn, Leu-Cr and Leu-Zn-Cr had better effect on the serum lipid profile than each supplement applied individually.

## Conclusions

Supplementary Cr(III) combined with Fe deficiency influenced the female rats’ Cu and Zn status.

Current findings show that Cr(III) supplementation alleviates the effects of Fe deficiency on Cu metabolism and deepens its effects on Zn metabolism.

Detailed data on trace element status and the direcions of their mutual interactions in healthy subjects as well as with the metabolic disorders (e.g. anaemia, diabetes mellitus) are strongly required for effective nutritional and therapeutic strategies.

## References

[1] Gutowska I, Machoy Z, Machoy-Mokrzynska A, Machalinski B (2009). The role of iron in metal-metal interactions in hard tissues of roe deer (*Capreolus capreolus* L.). Ann Acad Med Stetin.

[2] Chitturi R, Baddam VR, Prasad L (2015). A review on role of essential trace elements in health and disease. J NTR Univ Health Sci.

[3] Bjørklund G, Aaseth J, Skalny AV, Suliburska J, Skalnaya MG, Nikonorov AA, Tinkov AA (2017). Interactions of iron with manganese, zinc, chromium, and selenium as related to prophylaxis and treatment of iron deficiency. J Trace Elem Med Biol.

[4] Park E, Glei M, Knöbel Y, Pool-Zobel BL (2007). Blood mononucleocytes are sensitive to the DNA damaging effects of iron overload-in vitro and ex vivo results with human and rat cells. Mutat Res Fundam Mol Mech Mutagen.

[5] Sivasankari J, Thiruchelvan V (2017). Serum ferritin: an early marker of insulin resistance in metabolic syndrome. Int J Sci Study.

[6] Kulaksiz H, Fein E, Redecker P, Stremmel W, Adler G, Cetin Y (2008). Pancreatic β-cells express hepcidin, an iron-uptake regulatory peptide. J Endocrinol.

[7] Renuka P, Vasantha M (2016). Study of the serum levels of iron, ferritin and magnesium in diabetic complications. Int J Pharm Clin Res.

[8] Edgerton DS, Kraft G, Smith M, Farmer B, Williams PE, Coate KC, Printz RL, O’Brien RM, Cherrington AD (2017). Insulin’s direct hepatic effect explains the inhibition of glucose production caused by insulin secretion. JCI Insight.

[9] Cooksey RC, Jones D, Gabrielsen S, Huang J, Simcox JA, Luo B, Soesanto Y, Rienhoff H, Dale Abel E, McClain DA (2010). Dietary iron restriction or iron chelation protects from diabetes and loss of beta-cell function in the obese (ob/ob lep-/-) mouse. Am J Physiol Endocrinol Metab.

[10] Dogukan A, Sahin N, Tuzcu M, Juturu V, Orhan C, Onderci M, Komorowski J, Sahin K (2009). The effects of chromium histidinate on mineral status of serum and tissue in fat-fed and streptozotocin-treated type II diabetic rats. Biol Trace Elem Res.

[11] Basaki M, Saeb M, Nazifi S, Shamsaei HA (2012). Zinc, copper, iron, and chromium concentrations in young patients with type 2 diabetes mellitus. Biol Trace Elem Res.

[12] European Food Safety Authority (2014). Scientific opinion on dietary reference values for chromium. EFSA J.

[13] Vincent JB, Love S (2012). The binding and transport of alternative metals by transferrin. Biochim Biophys Acta.

[14] Quarles CD, Marcus RK, Brumaghim JL (2011). Competitive binding of Fe3+, Cr3+, and Ni2+ to transferrin. J Biol Inorg Chem.

[15] Quarles CD, Brumaghim JL, Marcus RK (2010). Instrumental comparison of the determination of Cr3+ uptake by human transferrin. Metallomics.

[16] Shah F, Kazi TG, Afridi HI, Kazi N, Baig JA, Shah AQ, Khan S, Kolachi NF, Wadhwa SK (2011). Evaluation of status of trace and toxic metals in biological samples (scalp hair, blood, and urine) of normal and anemic children of two age groups. Biol Trace Elem Res.

[17] Angelova MG, Petkova-Marinova TV, Pogorielov MV, Loboda AN, Nedkova-Kolarova VN, Bozhinova AN (2014). Trace element status (iron, zinc, copper, chromium, cobalt, and nickel) in iron-deficiency anaemia of children under 3 years. Anemia.

[18] Araya M, Pizarro F, Olivares M, Arredondo M, González M, Méndez M (2006). Understanding copper homeostasis in humans and copper effects on health. Biol Res.

[19] Osredkar J (2011) Copper and zinc, biological role and significance of copper/zinc imbalance. J Clin Toxicol. 10.4172/2161-0495.S3-001

[20] Chen T-S, Chen Y-T, Liu C-H, Sun CC, Mao FC (2015). Effect of chromium supplementation on element distribution in a mouse model of polycystic ovary syndrome. Biol Trace Elem Res.

[21] Hara T, Takeda T, Takagishi T (2017). Physiological roles of zinc transporters: molecular and genetic importance in zinc homeostasis. J Physiol Sci.

[22] Kaur K, Gupta R, Saraf SA, Saraf SK (2014). Zinc: the metal of life. Compr Rev Food Sci Food Saf.

[23] Tallman DL, Taylor CG (2003). Effects of dietary fat and zinc on adiposity, serum leptin and adipose fatty acid composition in C57BL/6J mice. J Nutr Biochem.

[24] Kaur S (2016). Iron deficiency anemia ( IDA ): a review. Int J Sci Res.

[25] Miranda ER, Dey CS (2004). Effect of chromium and zinc on insulin signaling in skeletal muscle cells. Biol Trace Elem Res.

[26] Nowak G (2015). Zinc, future mono/adjunctive therapy for depression: mechanisms of antidepressant action. Pharmacol Rep.

[27] Duce JA, Tsatsanis A, Cater MA, James SA, Robb E, Wikhe K, Leong SL, Perez K, Johanssen T, Greenough MA, Cho HH, Galatis D, Moir RD, Masters CL, McLean C, Tanzi RE, Cappai R, Barnham KJ, Ciccotosto GD, Rogers JT, Bush AI (2010). Iron-export ferroxidase activity of β-amyloid precursor protein is inhibited by zinc in Alzheimer’s disease. Cell.

[28] Vlizlo V, Iskra R, Maksymovych I (2014). Disturbance of antioxidant protection and natural resistance factors in rats with different availabilities of trivalent chromium. Turk J Vet Anim Sci.

[29] Earnshaw A, Figgis B, Lewis J (1966) Chemistry of polynuclear compounds. Part VI. Magnetic properties of trimeric chromium and iron carboxylates. J Chem Soc A Inorg Phys Theor :1656–1663. 10.1039/J19660001656

[30] Reeves PG (1997). Symposium : animal diets for nutritional and toxicological research. Exp Biol.

[31] McIver DJ, Grizales AM, Brownstein JS, Goldfine AB (2015). Risk of type 2 diabetes is lower in US adults taking chromium-containing supplements 1–3. J Nutr.

[32] Deng G, Wu K, Cruce AA, Bowman MK, Vincent JB (2015). Binding of trivalent chromium to serum transferrin is sufficiently rapid to be physiologically relevant. J Inorg Biochem.

[33] Deng G, Dyroff SL, Lockart M, Bowman MK, Vincent JB (2016). The effects of the glycation of transferrin on chromium binding and the transport and distribution of chromium in vivo. J Inorg Biochem.

[34] Bay A, Dogan M, Bulan K, Kaba S, Demir N, Öner AF (2013). A study on the effects of pica and iron-deficiency anemia on oxidative stress, antioxidant capacity and trace elements. Hum Exp Toxicol.

[35] Yokoi K, Kimura M, Itokawa Y (1991). Effect of dietary iron deficiency on mineral levels in tissues of rats. Biol Trace Elem Res.

[36] Staniek H, Wójciak RW (2018). The combined effect of supplementary Cr (III) propionate complex and iron deficiency on the chromium and iron status in female rats. J Trace Elem Med Biol.

[37] Staniek H, Wójciak RW (2018). The combined effects of iron excess in the diet and chromium (III) supplementation on the Iron and chromium status in female rats. Biol Trace Elem Res.

[38] EFSA NAD (2015). EFSA (panel on dietetic products nutrition and allergies) specific opinion on dietary values of chromium. EFSA J.

[39] Staniek H, Krejpcio Z (2017). The effects of supplementary Cr3 (chromium (III) propionate complex) on the mineral status in healthy female rats. Biol Trace Elem Res.

[40] Staniek H, Krejpcio Z (2009). The effects of tricentric chromium (III) propionate complex supplementation on pregnancy outcome and maternal and foetal mineral status in rat. Food Chem Toxicol.

[41] Staniek H, Rhodes NR, Di Bona KR (2013). Comparison of tissue metal concentrations in Zucker lean, Zucker obese, and Zucker diabetic fatty rats and the effects of chromium supplementation on tissue metal concentrations. Biol Trace Elem Res.

[42] Şahin K, Şahin N, Küçük O (2002). Effects of dietary chromium picolinate supplementation on serum and tissue mineral contents of laying Japanese quails. J Trace Elem Exp Med.

[43] Peng M, Yang X (2015). Controlling diabetes by chromium complexes: the role of the ligands. J Inorg Biochem.

[44] Sadri H, Larki NN, Kolahian S (2017). Hypoglycemic and hypolipidemic effects of leucine, zinc, and chromium, alone and in combination, in rats with type 2 diabetes. Biol Trace Elem Res.

[45] Racek J, Sindberg CD, Moesgaard S, Mainz J, Fabry J, Müller L, Rácová K (2013). Effect of chromium-enriched yeast on fasting plasma glucose , glycated haemoglobin and serum lipid levels in patients with type 2 diabetes mellitus treated with insulin. Biol Trace Elem Res.

[46] Tang HY, Xiao QG, Xu HB, Zhang Y (2015). Hypoglycemic activity and acute oral toxicity of chromium methionine complexes in mice. J Trace Elem Med Biol.

[47] Dȩbski B, Zalewski W, Gralak MA, Kosla T (2004). Chromium-yeast supplementation of chicken broilers in an industrial farming system. J Trace Elem Med Biol.

[48] Amatya JL, Haldar S, Ghosh TK (2004). Effects of chromium supplementation from inorganic and organic sources on nutrient utilization, mineral metabolism and meat quality in broiler chickens exposed to natural heat stress. Anim Sci.

[49] Dallago BSL, Lima BAF, Braz SV, Mustafa VS, McManus C, Paim TP, Campeche A, Gomes EF, Louvandini H (2016). Tissue accumulation and urinary excretion of Cr in chromium picolinate (CrPic)-supplemented lambs. J Trace Elem Med Biol.

[50] Liu Y, Hao P, Zhang X, Zhao X, Liu Y, Liu J (2016). Effects of excess Cr3+ on trace element contents in the brain and serum in chicken. Biol Trace Elem Res.

[51] Sahin K, Tuzcu M, Orhan C, Agca CA, Sahin N, Guvenc M, Krejpcio Z, Staniek H, Hayirli A (2011). The effects of chromium complex and level on glucose metabolism and memory acquisition in rats fed high-fat diet. Biol Trace Elem Res.

[52] Dubey VK, Ansari F, Vohora D, Khanam R (2015). Possible involvement of corticosterone and serotonin in antidepressant and antianxiety effects of chromium picolinate in chronic unpredictable mild stress induced depression and anxiety in rats. J Trace Elem Med Biol.

[53] Amani R, Saeidi S, Nazari Z, Nematpour S (2010). Correlation between dietary zinc intakes and its serum levels with depression scales in young female students. Biol Trace Elem Res.

[54] Dama M, Van Lieshout RJ, Mattina G, Steiner M (2018). Iron deficiency and risk of maternal depression in pregnancy: an observational study. J Obstet Gynaecol Can.

[55] Herrera C, Pettiglio MA, Bartnikas TB (2014). Investigating the role of transferrin in the distribution of iron, manganese, copper, and zinc. J Biol Inorg Chem.

[56] Lobo AR, Cocato ML (2010). Effects of inulin-type fructans consumption on mineral intestinal absorption and balance in rats fed control and iron-deficient diets. Alimetaria Nutr.

[57] Lobo AR, Gaievski EHS, De Carli E (2014). Fructo-oligosaccharides and iron bioavailability in anaemic rats: the effects on iron species distribution, ferroportin-1 expression, crypt bifurcation and crypt cell proliferation in the caecum. Br J Nutr.

[58] Tinkov AA, Ajsuvakova OP, Shehtman AM, Boev VM, Nikonorov AA (2012). Influence of iron and copper consumption on weight gain and oxidative stress in adipose tissue of Wistar rats. Interdiscip Toxicol.

[59] Tinkov AA, Polyakova VS, Nikonorov AA (2013). Chronic administration of iron and copper potentiates adipogenic effect of high fat diet in Wistar rats. BioMetals.

[60] Ścibior A, Zaporowska H (2007). Effects of vanadium(V) and/or chromium (III) on L-ascorbic acid and glutathione as well as iron, zinc, and copper levels in rat liver and kidney. J Toxicol Environ Health A.

[61] Tayrab E, Hamid A, Idriss HM (2013) Physiobiochemical metabolism serum copper and iron status in pregnant women with iron deficiency Anemia. p 2–4

[62] Bushra M, Elhassan EM, Ali NI, Osman E, Bakheit KH, Adam II (2010). Anaemia, zinc and copper deficiencies among pregnant women in central Sudan. Biol Trace Elem Res.

[63] McArdle HJ, Andersen HS, Jones H, Gambling L (2008). Copper and iron transport across the placenta: regulation and interactions. J Neuroendocrinol.

[64] Knovich MA, Il’yasova D, Ivanova A, Molnár I (2008). The association between serum copper and anaemia in the adult second National Health and nutrition examination survey (NHANES II) population. Br J Nutr.

[65] Wajeunnesa M, Begum N, Ferdousi S, Akhter S, Quaraishi SB (2009). Serum zinc and copper in iron deficient adolescents. J Bangladesh Soc Physiol.

[66] Turgut S, Hacıoğlu S, Emmungil G (2009). Relations between Iron deficiency anemia and serum levels of copper, zinc, cadmium and lead. Pol J Environ Stud.

[67] Ani M, Moshtaghie A (1992). The effect of chromium on parameters related to iron metabolism. Biol Trace Elem Res.

[68] Lukaski HC, Bolonchuk WW, Siders WA, Milne DB (1996). Chromium supplementation and resistance training: effects on body composition, strength, and trace elements status of men. Am J Clin Nutr.

[69] Lukaski HC, Siders WA, Penland JG (2007). Chromium picolinate supplementation in women: effects on body weight, composition, and iron status. Nutrition.

[70] Makhlough A, Makhlough M, Shokrzadeh M, Mohammadian M, Sedighi O, Faghihan M (2015). Comparing the levels of trace elements in patients with diabetic nephropathy and healthy individuals. Nephrourol Mon.

[71] Krisai P, Leib S, Aeschbacher S, Kofler T, Assadian M, Maseli A, Todd J, Estis J, Risch M, Risch L, Conen D (2016). Relationships of iron metabolism with insulin resistance and glucose levels in young and healthy adults. Eur J Intern Med.

[72] Skalnaya MG, Skalny AV, Tinkov AA (2017). Serum copper, zinc, and iron levels, and markers of carbohydrate metabolism in postmenopausal women with prediabetes and type 2 diabetes mellitus. J Trace Elem Med Biol.

[73] Ansar S, Alhefdhi T, Aleem AM (2015). Status of trace elements and antioxidants in premenopausal and postmenopausal phase of life: a comparative study. Int J Clin Exp Med.

[74] Gupta N, Arora KS (2011). The status of trace elements after menopause: a comparative study. J Clin Diagn Res.

[75] Vieyra-Reyes P, Millan-Aldaco D, Palomero-Rivero M (2016). An iron-deficient diet during development induces oxidative stress in relation to age and gender in Wistar rats. J Physiol Biochem.

[76] Sundaram B, Aggarwal A, Sandhir R (2013). Chromium picolinate attenuates hyperglycemia-induced oxidative stress in streptozotocin-induced diabetic rats. J Trace Elem Med Biol.

[77] Anderson RA, Roussel AM, Zouari N, Mahjoub S, Matheau JM, Kerkeni A (2001). Potential antioxidant effects of zinc and chromium supplementation in people with type 2 diabetes mellitus. J Am Coll Nutr.

